# Contribution of the uremic milieu to an increased pro-inflammatory monocytic phenotype in chronic kidney disease

**DOI:** 10.1038/s41598-019-46724-5

**Published:** 2019-07-15

**Authors:** Natalia Borges Bonan, Eva Schepers, Roberto Pecoits-Filho, Annemieke Dhondt, Anneleen Pletinck, Filip De Somer, Raymond Vanholder, Wim Van Biesen, Andréa Moreno-Amaral, Griet Glorieux

**Affiliations:** 1 Pontifícia Universidade Católica do Paraná, Curitiba, Brazil; 2 Ghent University Hospital, Nephrology Division, Gent, Belgium; 3 Ghent University Hospital, Heart Centre, Gent, Belgium

**Keywords:** Cell biology, Nephrology

## Abstract

Intermediate (CD14^++^CD16^+^) monocytes have important pro-inflammatory and atherogenic features and are increased in patients with chronic kidney disease (CKD). The present study aims to elucidate the role of the uremic milieu and of platelet activation in monocyte differentiation. Monocyte subtypes were analyzed in CKD patients (n = 193) and healthy controls (n = 27). Blood from healthy controls (Ctrl; n = 8) and hemodialysis patients (HD; n = 8) was centrifuged, and plasma (pl) was exchanged between Ctrl and HD (Ctrlcells/HDpl and HDcells/Ctrlpl) or reconstituted as original (Ctrlsham and HDsham) and incubated for 24 h (T24). Monocyte differentiation and platelet aggregation to monocytes (MPA) was assessed by flow cytometry. Especially, a higher proportion of CD14^++^CD16^+^ monocytes was found in hemodialysis (HD) patients (p < 0.01). In plasma exchange experiments, Ctrl cells/HD pl T24 showed an increased percentage of CD14^++^CD16^+^ monocytes versus Ctrl sham (33.7% ± 15 vs. 15.7% ± 9.6; P < 0.005), comparable to the level of CD14^++^CD16^+^ monocytes in the HD sham condition. The percentage of CD14^++^CD16^+^ monocytes was lowered by suspending HD cells in Ctrl pl (18.4% ± 7.8 vs. 36.7% ± 15 in HD sham; P < 0.005) reaching the level of the Ctrl sham condition (15.7% ± 9.6). A mixture of uremic sulfates increased CD14^++^CD16^+^ monocytes compared to control (19.8 ± 9.6% vs. 15.8 ± 10.9%; P < 0.05), paralleled by a rise MPA. Blocking MPA by abciximab, a potential therapeutic strategy, or anti-CD62P did not inhibit differentiation towards the CD14^++^CD16^+^ monocytes. In conclusion, in the present cohort, CD14^++^CD16^+^ monocytes are especially increased in HD patients and this can at least in part be attributed to the presence of the uremic milieu, with uremic sulfates inducing a reversible shift towards pro-inflammatory CD14^++^CD16^+^ monocytes.

## Introduction

Chronic kidney disease (CKD) is a global health issue due to its high prevalence and its association with an increased risk for atherosclerotic cardiovascular disease (CVD), a major cause of death in this population^1,2^. Vascular damage is initiated by endothelial dysfunction and monocyte activation. Monocytes play a crucial role in the innate immune system homeostasis, immune defense, tissue repair and in the development of atherosclerosis^3^. Additionally, activated platelets are involved in this process promoting aggregates with monocytes and also in plaque rupture that leads to ischemic events^4^.

According to the International Union of Immunological Societies (IUIS) peripheral monocytes can be classified into three distinct subpopulations based on their expression of CD14 (differentiation antigen and lipopolysaccharide receptor) and CD16 (low affinity Fc receptor): classical (CD14^++^CD16^−^), intermediate (CD14^++^CD16^+^) and non-classical monocytes (CD14^+^CD16^++^)^5^. CKD patients and hemodialysis (HD) patients have a higher percentage of intermediate monocytes (CD14^++^CD16^+^) in the circulation compared to healthy subjects^6,7^ and this specific monocyte subtype has been associated with increased cardiovascular events and progression of anemia in CKD^8,9^.

Uremia has been related to increasing leukocyte activity and inflammation, which can in part be attributed to the accumulation of uremic toxins. *p*-Cresyl sulfate (pCS) was already reported to induce increased oxidative burst activity of monocytes at baseline^10^. Also, pro-inflammatory cytokines which are found at increased levels in uremic patients, such as TNF-α, are able to induce reactive oxygen species (ROS) production in monocytes^11^. In addition, the accumulation of other uremic toxins such as homocysteine, which may trigger epigenetic dysregulation in circulating cells, induces monocyte differentiation and activation of their pro-inflammatory signaling cascades, which plays a role in ROS metabolism^12^. Symmetric dimethylarginine (SDMA) showed an activation of Nuclear Factor κB, resulting in an increased expression of IL-6 and TNF-α^13^. A comprehensive overview of uremic toxins with reportedly pro-inflammatory properties can be found in^14^.

Monocyte differentiation is a gradual process in which CD14^++^CD16^−^ monocytes differentiate into CD14^++^CD16^+^ monocytes and CD14^+^CD16^++^ monocytes permitting these cells to adapt to the milieu^15^. Philips *et al*. suggested an involvement of activated platelets in increased CD16 expression by monocytes^16^ and it was also shown that, in healthy subjects post *Influenza* immunization, the subsequent increased interaction between monocytes and platelets induces an increase in the percentage of CD16 positive monocytes^17^. The primary adhesion molecule for this interaction is p-selectin (CD62P), which is expressed by activated platelets^18^ and is suggested to be related to the induction of CD16 expression^19^.

In the present study, the proportion of monocyte subpopulations was determined in a cross-sectional CKD cohort. In a second step, the role of the uremic milieu and the involvement of platelet activation in the differentiation process of the monocytes was evaluated.

## Results

### Monocyte subtypes in CKD and different dialysis modalities

**Figure 1:**
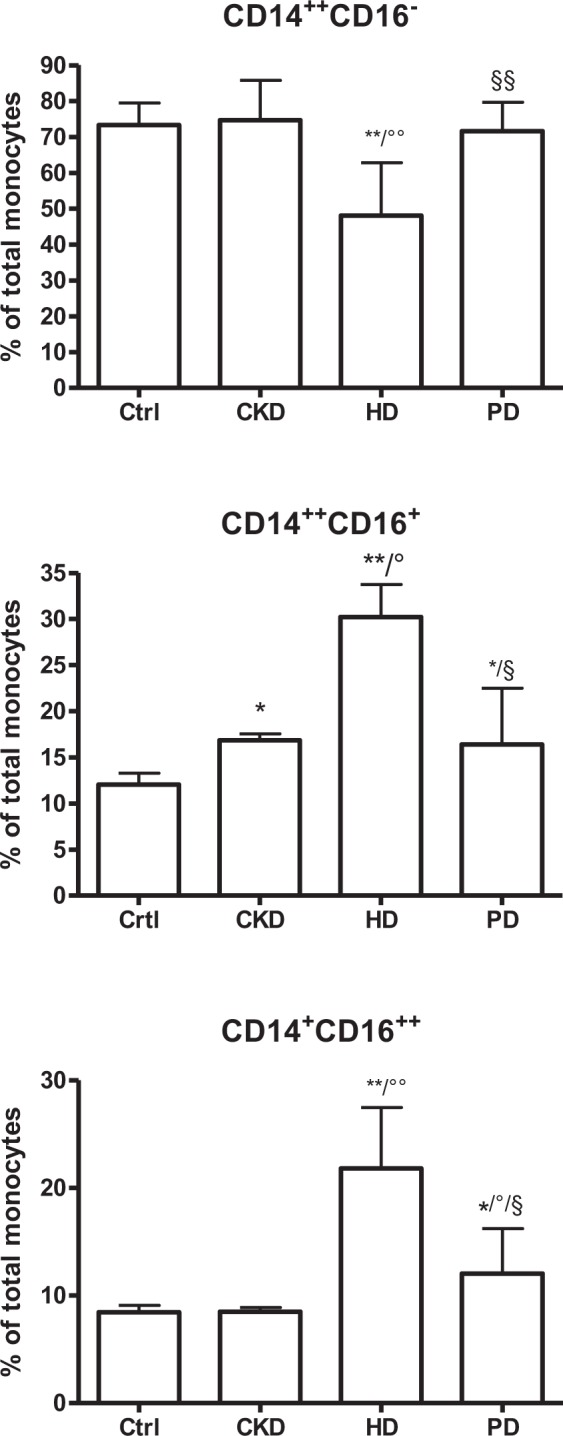
Monocyte subpopulations in CKD patients versus healthy control (Ctrl). CKD: patients with chronic kidney disease stages 1 to 5; HD: hemodialysis patients; PD: peritoneal dialysis patients. Results are presented a mean ± SD. One significance symbol: p < 0.05, two symbols: p < 0.01; * vs. Ctrl; ° vs. CKD; § vs. HD. CD14^++^CD16^−^: classical monocytes; CD14^++^CD16^+^: intermediate monocytes; CD14^+^CD16^++^: non classical monocytes.

Patient characteristics are summarized in Table 1. Patients match well for gender, age, body mass index (BMI) while creatinine and urea is higher and total protein lower in patients on dialysis. The frequency of diabetics is comparable in patients on dialysis or not. Also total cholesterol, HLD cholesterol and total triglycerides are comparable among the 3 CKD groups. The use lipid lowering drugs and anti-aggregates are reported for the CKD patients not on dialysis. Total leukocyte and monocyte count do not differ among the different groups. Figure 1 shows the distribution of the monocytes subtypes in CKD patients on dialysis or not. The percentage of CD14^++^CD16^+^ monocytes is slightly, but significantly, higher in CKD and PD patients (p < 0.05) and more than twofold higher in patients on hemodialysis (HD) vs. healthy control (Ctrl) (p < 0.01) with a corresponding decrease in CD14^++^CD16^−^ (p < 0.01) and increase in CD14^+^CD16^++^ monocytes in HD (P < 0.01). However, no negative correlation was found between eGFR and the percentage of intermediate monocytes in CKD patients not on dialysis (ρ = 0.157; p = 0.039) (Supplementary Fig. 1). Change in percentage of monocyte subtypes are also reflected in changes in their absolute numbers (data not shown). The latter observation is important when estimating the total inflammatory capacity of the monocytes in CKD patients compared to healthy subjects.

**Table 1:** Patient characteristics for enummeration experiments.

	CKD1-5	HD	PD	P-value
Number	175	7	11	
Age (y)	63.3 (26.6)	68.0 (24.0)	50.0 (21.0)	NS
Gender (M/F)	116/59	3/4	9/2	NS
BMI (kg/m²)	27.9 (7.1)	22.6 (15.5)	26.0 (3.3)	NS
Diabetes (%)	34.3	40.0	33.3	NS
Creatinine (mg/dL)	1.4 (0.7)	8.1 (3.2)	6.6 (3.9)	0.0001
Urea (mg/dL)	0.52 (0.45)	na	1.21 (0.13)	0.005
Serum protein content (g/L)	70.1 ± 0.5	65.0 ± 8.0	59.0 ± 4.9	0.032
Total cholesterol (mg/dL)	180.3 (54.2)	159.0 (48.0)	170.0 (56.7)	NS
HDL cholesterol (mg/dL)	50.9 (21.0)	46.0 (5.0)	52.5 (25.4)	NS
Fasting triglycerides (mg/dL)	127.2 (78.3)	134.0 (64.0)	136.0 (64.6)	NS
CRP (mg/L)	1.8 (3.2)	9.0 (10.0)	3.5 (9.6)	NS
WBC (/µl)	7071 (2876)	6813 (2857)	6735 (2915)	NS
Monocytes (/µl)	768 (379)	948.8 (1270.5)	640.5 (634.4)	NS
Ezetimibe (%)	0.6	na	na	
Fibrates (%)	2.2	na	na	
Statins (%)	55.6	na	na	
Aspirin (%)	33.7	na	na	
Other anti-aggregates (%)	9	na	na	

Abbreviations: CKD: Chronic kidney disease; HD: hemodialysis; PD: peritoneal dialysis; BMI: body mass index; CRP: C-reactive protein; WBC; White blood cells; na: not available; NS: not significant. Data are reported as mean ± SD or median (interquartile range).

### Monocyte subpopulations and monocyte platelet aggregate formation in healthy donors versus HD patients at baseline

**Figure 2:**
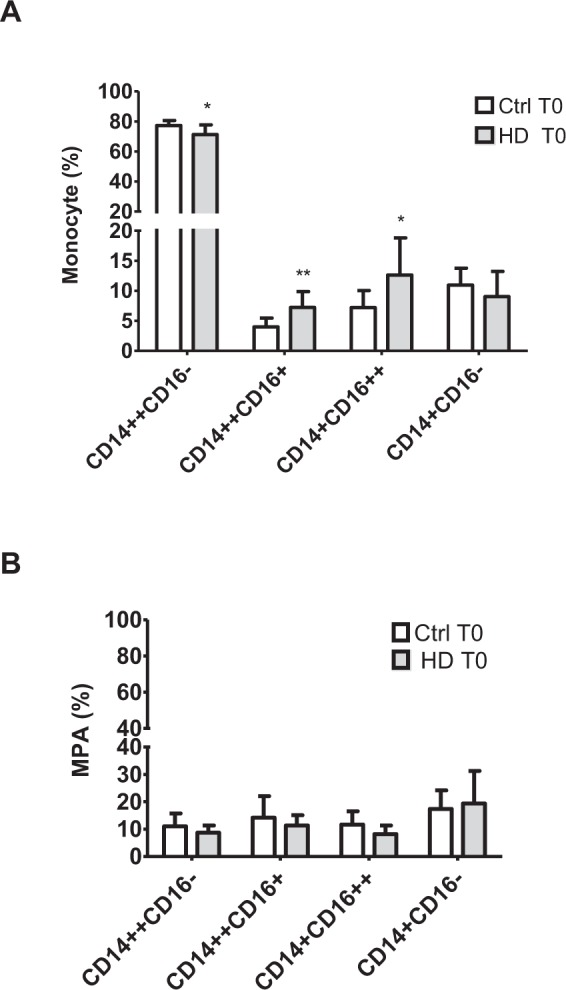
Monocyte subpopulations (**A**) and monocyte subpopulation-platelet aggregates (**B**) in healthy controls (Ctrl; white bars) and HD patients (grey bars) immediately after collection. Sodium citrate blood from healthy control (Ctrl; n = 8) and hemodialysis patients (HD; n = 8) Results are presented a mean ± SD. *p < 0.05, **p < 0.005 vs. Ctrl. CD14^++^CD16^−^: classical monocytes; CD14^++^CD16^+^: intermediate monocytes; CD14^+^CD16^++^: non classical monocytes; CD14^+^CD16^−^: negatives; MPA: monocyte-platelet aggregates.

Donor characteristics are shown in Table 2. Figure 2A illustrates the distribution of monocyte subpopulations in whole blood of healthy donors and HD patients immediately after collection. In parallel to a lower percentage of CD14^++^CD16^−^ monocytes (71.3% ± 6.5 vs. 77.3% ± 3.4; P < 0.05), a corresponding higher percentage of CD14^++^CD16^+^ monocytes (7.2% ± 2.6 vs. 4.0% ± 1.4; P < 0.005) and CD14^+^CD16^++^ monocytes (12.6% ± 6.2 vs. 7.2% ± 2.8; P < 0.05) was observed in HD patients compared to control (Ctrl). In contrast to a moderate, but significant increase in the percentage of activated platelets (CD62 positivity) in HD patients vs. control (2.3%[14.6] vs.1.1%[7.8]; p < 0.05) no difference in monocyte platelet aggregates (MPA) for any of the monocyte subtypes was observed (Fig. 2B).

**Table 2:** Patient characteristics for exchange experiments.

Characteristics	Controls (n = 8)	HD (n = 8)
Age, years	42.8 ± 12.8	70.8 ± 19.7*
Gender (M/F)	0/8	6/2
BMI (kg/m²)	22.2 ± 2.5	23.0 ± 4.2
Creatinine (mg/dL)	0.8 ± 0.1	7.2 ± 1.5*
Serum protein content (g/L)	68.8 ± 2.3	65.5 ± 5.3
CRP (mg/L)	1.4 ± 2.9	5.0 ± 6.6
WBC (/µl)	588 ± 153″	5522 ± 154
Monocytes (/µl)	506 ± 124.4	427 ± 247

Data reported are mean ± SD. *p < 0.005 vs. control. Abbreviations: CRP: C-reactive protein, WBC: white blood cells; HD: hemodialysis; BMI: body mass index.

### Effect of the uremic milieu on monocyte differentiation and MPA formation after plasma exchange

**Figure 3:**
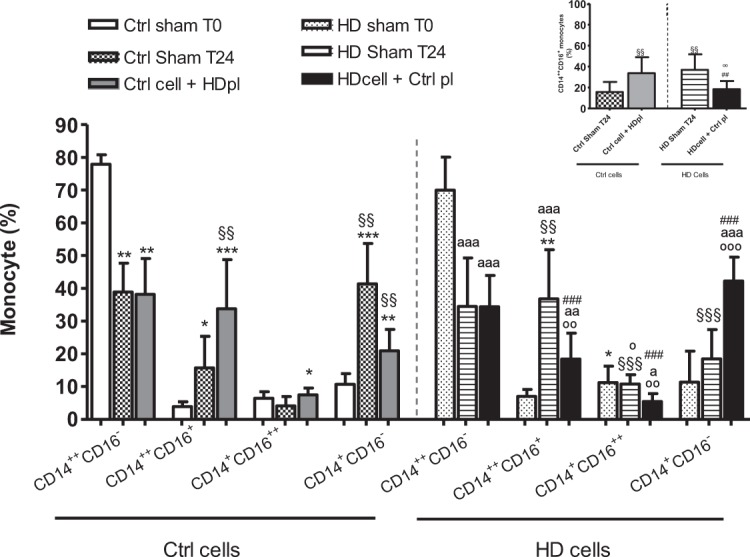
Effect of the uremic milieu (HD plasma (pl)) on healthy monocyte subpopulations compared to the effect of the healthy milieu (Ctrl plasma (pl)) on HD monocyte subpopulations. Sodium citrate blood from healthy controls (Ctrl) (n = 8) and hemodialysis patients (HD; n = 8) was centrifuged and the autologous plasma was completely removed and re-added (sham) or exchanged for blood type matched HD plasma and vice versa and incubated for 24 h. Data express monocytes subpopulations (%); upper right: zooms in on intermediate monocytes (CD14^++^CD16^+^). Results are presented a mean ± SD. One significance symbol: p < 0.05, two symbols: p < 0.005 and three symbols: p < 0.001. *vs. Ctrl sham T0; ^§§^vs. Ctrl sham T24; °vs. Ctrl cell + HD pl; ªvs. HD sham T0; ^#^vs. HD sham T24.

Sham treatment of the plasma did not change the distribution of the monocyte subpopulations (data not shown). After 24 h incubation (Ctrl sham T24), the percentage of CD14^++^CD16^−^ monocytes decreased by 50% with a corresponding 5-fold increase in the percentage of CD14^++^CD16^+^ monocytes (15.7% ± 9.6 vs. 3.9% ± 1.42; P < 0.005) and in the percentage of CD14^+^CD16^−^ monocytes compared to baseline (Ctrl sham T0) (Fig. 3). When healthy cells were incubated for 24 h in the presence of HD plasma (Ctrl cell + HD pl T24), the percentage of CD14^++^CD16^+^ monocytes additionally doubled (33.7% ± 15.0; P < 0.005) and the percentage of CD14^+^CD16^++^ increased (7.5% ± 2.1 vs. 4.1% ± 2.7; P < 0.005) in comparison to the Ctrl sham T24 condition (Fig. 3) In parallel, HD plasma induced an increase in the percentage of CD14^++^CD16^−^ monocytes adhering to platelets (MPA) compared to control sham T24 (25.7% ± 13.6 vs. 20.3% ± 7.2; P < 0.05). For the other subtypes no significant difference in aggregation with platelets was observed (data not shown).

Interestingly, on the zoom graph of Fig. 3 reversibility of the uremic effect was highlighted since the increased percentage of CD14^++^CD16^+^ monocytes (36.8% ± 15.0) observed in HD patients at T24 (HD sham T24) was lower after a 24 h incubation in the presence of the exchanged control plasma (HD cell + Ctrl pl T24) (18.4% ± 7.8; P < 0.0005). The latter coincided with a higher percentage of CD14^+^CD16^-^ monocytes compared to HD sham T24 (42.3% ± 7.3 vs. 18.6% ± 8.7; P < 0.0001). Figure 3, focusing on the CD14^++^CD16^+^ monocytes, demonstrates that incubating healthy monocytes with HD plasma results in a comparable proportion of this pro-inflammatory subtype as present in HD sham blood after 24 h incubation. When HD plasma is replaced by healthy plasma the proportion of CD14^++^CD16^+^ monocytes is comparable to the control sham condition after 24 h. The changes in percentage of monocyte subtypes are paralleled by  changes in their absolute numbers (data not shown).

### Effect of uremic toxins on monocyte differentiation and MPA formation

**Figure 4:**
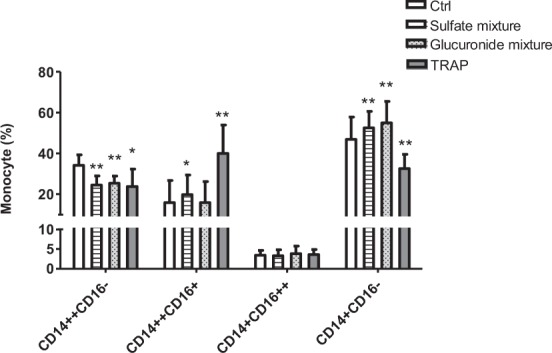
Effect of uremic toxins on monocyte differentiation. Sodium citrate blood from healthy donors (n = 8) was incubated for 24 h at 37 °C in the presence of the salt control solution (Ctrl), sulfate mixture [indoxyl sulfate (IxS), p-cresyl sulfate (pCS), phenyl sulfate (PhS); sulfates], glucuronide mixture [indoxyl glucuronide (IG), p-cresyl glucuronide (pCG), phenyl glucuronide (PhG); glucuronides] or thrombine receptor activator peptide (TRAP). Data express the percentage of monocyte subtypes. Results are presented a mean ± SD. *p < 0.05; **p < 0.0005 vs. Ctrl.

**Figure 5:**
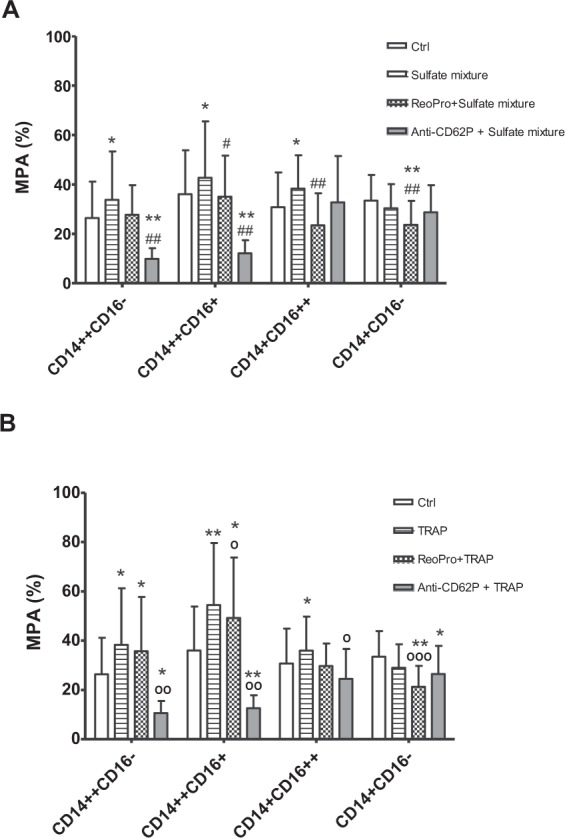
Effect of uremic toxins on monocyte subpopulation-platelet aggregation (%). Sodium citrate blood from healthy donors (n = 8) was pre-incubated in the absence and presence of abciximab or anti-CD62P, and incubated for 24 h in the presence of salt control solution (Ctrl), (**A**) sulfate mixture or (**B**) thrombine receptor activator peptide (TRAP). Results are presented a mean ± SD. *p < 0.05 vs. Ctrl; ^#/°^p < 0.05 vs. sulfates/TRAP; ^##/°°^p < 0.005 vs. sulfates/TRAP; °°°p < 0.0005 vs. TRAP. MPA: monocyte-platelet aggregates. CD14^++^CD16^-^: classical monocyte-platelet aggregates; CD14^++^CD16^+^: intermediate monocyte-platelet aggregates; CD14^+^CD16^++^: non classical monocyte-platelet aggregates; CD14^+^CD16^−^: CD14^+^CD16^–^platelet aggregates.

Figure 4 shows the monocytes subtypes after a 24 h incubation of healthy cells in the presence of a sulfate [indoxyl sulfate (IxS), p-cresyl sulfate (pCS), phenyl sulfate (PhS)] mixture or a glucuronide [indoxyl glucuronide (IxG), p-cresyl glucuronide (pCG), phenyl glucuronide (PhG)] mixture. Incubation in the presence of the sulfate mixture resulted in an increase of the percentage of the pro-inflammatory CD14^++^CD16^+^ monocytes compared to control (19.8 ± 9.6% vs. 15.8 ± 10.9%; P < 0.05). Thrombine receptor activator peptide (TRAP), used as a positive control for platelet activation, also induced differentiation towards the CD14^++^CD16^+^ monocytes (40.0 ± 14.0% vs. 15.8 ± 10.9%; P < 0.005) compared to control. No such effect was observed in the presence of the glucuronide mixture. Both the mixture of sulfate and of glucuronide resulted in about 10% less CD14^++^CD16^−^ monocytes and a higher percentage of CD14^+^CD16^−^ monocytes vs. the control condition. In addition, the sulfate mixture, like TRAP, induced more platelet aggregation to the first three monocyte subtypes compared to the control condition (Fig. 5A,B). The glucuronide mixture did not induce an increase in MPA (data not shown). Anti-CD62P was shown to prevent aggregation of platelets to CD14^++^CD16^−^ and CD14^++^CD16^+^ monocytes induced by the sulfate mixture or to CD14^++^CD16^−^, CD14^++^CD16^+^ and CD14^+^CD16^++^ monocytes induced by TRAP. However, this inhibition in MPA did not prevent differentiation towards CD14^++^CD16^+^ monocyte subtype (data not shown). In contrast, inhibition of MPA induced by abciximab was less pronounced or even absent. Abciximab also did not prevent monocyte differentiation towards the CD14^++^CD16^+^ monocyte subtype.

## Discussion

This study showed, especially in HD patients, increased levels of pro-inflammatory CD14^++^CD16^+^ monocytes, which is in line with previous findings in HD and non-dialysis CKD patients^9,20,21^. Next, the effect of the uremic milieu and specific uremic toxins on monocyte differentiation and the possible role of platelet aggregation on monocyte differentiation were investigated in HD patients. It was shown that after 24 h incubation the uremic milieu compared to control plasma promoted an increase in CD14^++^CD16^+^monocytes. This effect of the uremic milieu was reversible as demonstrated by incubating uremic cells in healthy plasma. In parallel, a mixture of sulfate conjugates containing IxS, pCS and PhS induced differentiation towards the intermediate (CD14^++^CD16^+^) phenotype. Although the platelet activator TRAP was shown to induce a differentiation towards the CD14^++^CD16^+^ monocytes, no effect was observed with respect to platelet aggregation *per se*.

Monocytes are considered to play a role in the micro-inflammation of CKD and this could, at least in part, be attributed to a higher proportion of CD14^++^CD16^+^ cells in the monocyte population, as also observed in other inflammatory diseases such as asthma and rheumatoid arthritis^22,23^. The pro-inflammatory character of this phenotype is supported by their increased expression of pro-inflammatory cytokines, such as TNF-α and IL-6, and their increased capacity to produce reactive oxygen species^24,25^ which are both undisputable patho-physiologic characteristics of the uremic syndrome^26^. The decreased percentage of CD14^++^CD16^−^ monocytes in parallel to an increased percentage of CD14^++^CD16^+^ and CD14^+^CD16^++^ monocytes in HD patients compared to controls (Fig. 1) suggests that CD14^++^CD16^−^ monocytes differentiate toward CD14^++^CD16^+^ and CD14^+^CD16^++^^15^. Remarkably, the present study also revealed a CD86-positive monocyte subtype which was low in CD14 and without expression of CD16 (named CD14^+^CD16^−^ monocytes). Although these cells constitute a substantial fraction of the total monocyte population and were confirmed to be monocytes based on their CD86 positivity^27,28^, in this study no functional impact of this subtype was explored. Remarkably, CD14^++^CD16^+^ monocytes are only slightly higher in PD patients vs. controls. This observation is in line with the findings of Merino *et al*. who found a relationship between CD14^++^CD16^+^ monocytes and endothelial damage in HD patients but not in PD patients^20,29^. It is of note that the relevance of the increased percentage of CD14^++^CD16^+^ monocytes in the present CKD group not on dialysis (Fig. 1) is questionable in view of the absence of a negative correlation between eGFR and the percentage of CD14^++^CD16^+^ monocytes (Supplementary Fig. 1). Based on our results the relevance appears to confined to the HD patients.

Inflammation is considered to contribute to the cardiovascular risk of CKD and the expanded number of pro-inflammatory monocytes has been related to the inflammation induced by uremia^30^. Monocytes and platelets are known to be associated to atherosclerosis and cardiovascular events^31^. CD14^++^CD16^+^ monocytes have already been associated to these comorbidities of CKD^7,32^. Uremic serum was already shown to induce monocyte differentiation^9^ and in the present study at 24 h incubation of healthy cells with HD plasma the percentage of CD14^++^CD16^+^ monocytes was higher while the percentage of CD14^++^CD16^−^ monocytes was lower vs. incubation with control plasma. This suggests that plasma from HD patients can induce differentiation from the CD14^++^CD16^−^ towards the intermediate phenotype. Removal of the uremic milieu, as mimicked by replacing HD plasma with healthy plasma, could reverse monocyte differentiation and thus reduce this pro-inflammatory phenotype. Of note, changes in monocyte profile in the present CKD cohort could not have been influenced by the presence of diabetes^33^ or a perturbed lipid profile^34^ since the frequency of diabetes and the lipid levels were comparable among the different groups (Table 1).

While the proportion of CD14^++^CD16^−^ monocytes remained the same, the CD14^+^CD16^−^ subpopulation increased. This result suggests that the cell phenotype changes or adapts, depending on the milieu. It also seems that once the monocytes are activated and start to differentiate they continue along. However, extra assays are needed to confirm this sequence of events. Comparable to the effect of HD plasma, sulfates (pCS, IxS, PhS) were able to promote monocyte differentiation, indicating the contribution of uremic toxins to a shift in monocyte subpopulations, as previously demonstrated for homocysteine which induced epigenetic dysregulation which affected several transcription regulators important for monocyte differentiation [e.g., fms-like tyrosine kinase 3 (FLT3); Histone deacetylase 1 (HDAC1); MAX network transcriptional repressor (MNT)] leading to enhanced generation of CD14^++^CD16^+^ monocytes^12^.

The role of platelets in CKD is still controversial. Platelets have traditionally been described to be involved in maintenance of hemostasis but more recent data pointed to their role in inflammation and immune response^35^. Our data showed that HD patients presented a moderate, but significantly higher percentage of activated platelets, compared to controls. There is evidence that platelets play an important role in the induction of CD16 expression in monocytes preceded by early release of TGF-β^16^. An *in vitro* study, using influenza infection to promote inflammatory response, showed the importance of platelet activation and monocyte-platelet aggregation in the increased percentage of CD16 positive monocytes in the circulation, mainly driven by expansion of the intermediate subpopulation^17^. The possible role of platelets in monocyte differentiation was also supported in this study by our positive control experiments with TRAP, which induced an increase in platelet aggregates to all monocyte subpopulations, whereby the aggregation to especially classical (CD14^++^CD16^−^) monocytes might initiate the differentiation pathway.

We also evaluated whether blocking MPA could affect differentiation of monocytes. CD62P is a p-selectin stored in the α-granules or expressed on the surface of activated platelets and it is an essential molecule for MPA formation^36^. It could be expected that, by adding a CD62P antibody or abciximab, an antibody against glycoprotein IIb/IIIa expressed on the platelets which is also involved in monocyte-platelet aggregation, MPA formation could at least in part be blocked^37^. A previous study showed that co-incubation of monocytes with anti-PSGL-1, (P-selectin glycoprotein-ligand-1 which is constitutively expressed in leukocytes), was able to block MPA and also presented a reduced degree of induction of CD16 on those monocytes^17^. The present study suggests that adhesion induced by uremic toxins or TRAP primarily occurs via CD62, however this is not the trigger for monocyte differentiation since it was not possible to block the differentiation by blocking MPA via CD62P nor glycoprotein IIb/IIIa. Very likely it would be necessary to block more than one receptor on platelets to have an effect in monocytic CD16 expression since platelets and monocytes reportedly interact via many molecular pathways and mediators, such as CD40L, TREM-1 ligand^36^, or TGF-β. The latter, present in α-granules of activated platelets is known to interfere with leukocyte immune response^16,17,19,36,37^.

In summary, intermediate (CD14^++^CD16^+^) monocytes are only slightly elevated in CKD and PD patients while in hemodialysis patients more than a doubling in CD14^++^CD16^+^ monocytes was observed compared to healthy controls. Plasma from HD patients induced differentiation of healthy monocytes towards the intermediate phenotype. This process was reversed by replacing the uremic milieu with healthy plasma. The combination of IxS, pCS and PhS also presented an increase in the proportion of in pro-inflammatory CD14^++^CD16^+^ monocytes. However, blocking monocyte-platelet aggregation with anti-CD62 and anti-glycoprotein IIb/IIIa alone was insufficient to decrease monocyte differentiation towards the pro-inflammatory phenotype.

## Material and Methods

### Whole blood samples

For the *ex vivo* quantification of monocyte subtypes, 193 patients at different stages of CKD including patients on HD and PD were recruited in the Nephrology outpatient clinic (Ghent University Hospital, Belgium) and were compared to a healthy control group (n = 27). The creatinine-based Chronic Kidney Disease Epidemiology Collaboration-formula (CKD-epi) was used to estimate GFR and the CKD stage were classified as defined by the National Kidney Foundation’s Kidney Disease Outcomes Quality Initiative (KDOQI)^38^. Blood was collected in K_2_EDTA tubes (Vacutainer^®^ tubes, Becton Dickinson, Plymouth, UK) after antecubital venipuncture with a butterfly needle. Counts were performed immediately after collection.

For the *in vitro* differentiation experiments clinically stable HD patients from the hemodialysis unit (Ghent University Hospital, Belgium) and healthy volunteers were recruited. HD patients were on thrice-weekly post-dilution hemodiafiltration for at least 3 months. Exclusion criteria were: presence of acute or chronic infection, active immunological disease, use of immunosuppressive or anti-inflammatory drugs within the last 3 months and active smoking. For HD patients, blood was drawn before hemodialysis from the fistula or catheter. For healthy donors, blood samples were drawn by antecubital venipuncture using a 20Gx1 1/2″ UTW needle (0.9 × 40 mm) (Terumo Europe N.V, Leuven, Belgium) to minimize platelet activation during venipuncture. Blood was collected in an K_2_EDTA tube (Vacutainer^®^ tubes, Becton Dickinson, Plymouth, UK) and sodium citrate Vacutainer^®^ tubes (Becton Dickinson, Plymouth, UK). The first collected K_2_EDTA tube was used to quantify creatinine, total protein, C-reactive protein (CRP) and urea as routine parameters measured by standard techniques, and not for the incubation experiments.

### Ethics statement

This study was approved by the Medical Ethics Committee of the Ghent University Hospital (Ghent, Belgium) (B67020107926-2010/033 and B670201629781-2016/1180). Written informed consent was obtained from each participant prior to sample collection. All experiments were performed in accordance with the WMA declaration of Helsinki (1964) and its later amendments on ethical principles for medical research involving human subjects.

### Antibodies and reagents

The antibodies against CD14-PerCP, CD16-PECy7, CD86- APC, CD61–FITC, CD62P-PE, unlabeled CD62P and FACS™-Lysing solution were obtained from BD Bioscience (San Jose, CA, USA). Thrombin receptor activator peptide (TRAP – 10 µM) was purchased from Sigma Aldrich (St Louis, MO) and abciximab [an antibody against glycoprotein IIb/IIIa inhibiting platelet aggregation (ReoPro®; 2 mg/ml)] from Lilly Benelux S.A/N.V.

### Uremic toxins

*p-*Cresyl sulfate and phenyl sulfate were synthesized as a potassium salt according to Feigenbaum and Neuberg^39^ and *p-*Cresyl glucuronide from protected glucuronyl-trichloroacetimidate and *p*-cresol using a protocol similar to the one described by Van der Eycken *et al*.^40^ (laboratory for organic and Bio-organic Synthesis (Ghent University, Belgium). Phenyl-β-D-glucuronide, indoxyl-β-D-glucuronide cyclohexylammonium salt and indoxyl sulfate potassium salt were purchased from Sigma Aldrich (St Louis, MO). The individual compounds were prepared in saline (NaCl 0.9%, B. Braun, Melsungen; Germany) as 60x stock solutions. A mixture of sulfates (pCS, PhS and IxS) and a mixture of glucuronides (pCG, PhG and IxG) was composed as a 10x stock solution. Stock solution of KCl and/or NH_4_Cl in saline were prepared to be used as a salt control. All solutions were aliquoted and stored at −80 °C until needed. For each experiment the 10x stock solutions were added to whole blood (1:10) to obtain a representative uremic concentration for each toxin: IxS:44.5 mg/L; pCS: 43.0 mg/L; IxG: 3.9 mg/L; PhS: 13.5 mg/L; PhG:1.6 mg/L; pCG: 7.3 mg/L^41–44^.

### Immunolabeling and flow cytometry

**Figure 6:**
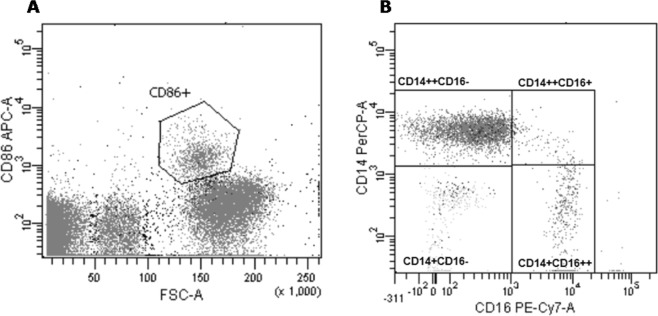
Representative plot illustrating identification of monocyte subpopulations. (**A**) Gating of monocytes based on CD86 positivity and (**B**) gating of monocyte subpopulations based on CD14 and CD16 positivity.

Whole blood (100 µl) was stained immediately after collection, after plasma exchange or sham treatment and after 24 h incubation (T24) with the antibodies mentioned above (25 minutes (min) at room temperature (RT) in the dark). Next, leukocytes were fixed and erythrocytes lysed by FACS™-lysing solution. After washing (5 min at 390 *g*), samples were analyzed by flow cytometry (FACSCanto™, BD Bioscience) using the FACSDIVA™ software. Leukocytes were identified based on forward (FSC) and side scatter (SSC) properties. Monocytes were identified by gating on the PAN monocytic marker CD86 (Fig. 6A) and the subpopulations of monocytes were distinguished by their CD14 and CD16 expression as classical (CD14^++^CD16^−^), intermediate (CD14^++^CD16^+^), non-classical (CD14^+^CD16^++^) and CD14^+^CD16^−^ population (Fig. 6B).

Platelets were identified based on their CD61 expression and platelet activation was evaluated by CD62P positivity. The monocyte-platelet aggregates were identified per monocyte subpopulation as those monocytes which were also positive for CD61 or CD62P.

### Effect of the total uremic milieu on monocyte differentiation and MPA formation

Healthy blood donors (n = 8) and HD patients (n = 8) were matched for blood group and rhesus factor to limit activation due to blood type incompatibility. To analyze the effect of the total uremic milieu on monocyte differentiation, both healthy blood and HD blood were centrifuged, the autologous plasma was completely removed and re-added (sham) or exchanged for HD plasma and vice versa. After careful mixing, whole blood was incubated under static conditions in a multi-well plate (Nunclon® Thermo Scientific, Roskilde, Denmark) for 24 h at 37 °C, 95% humidity and 5% CO_2_. Immediately after plasma exchange (T0) and after incubation (T24), cells were labeled for CD14, CD16, CD86, CD61 and CD62P and analyzed by flow cytometry as described above.

### Effect of specific uremic toxins on monocyte differentiation and MPA formation

Whole blood from healthy donors (n = 8) was incubated for 24 h in the absence (salt control) and presence of uremic toxins (sulfate and glucuronide mixture) and TRAP (2 µM) was used as a positive control for platelet activation. Anti CD62P (6.25 µg/mL) an inhibitor of platelet activation, was added 45 min before adding toxins or TRAP. After 24 h incubation samples were labeled and analyzed as described above.

### Statistical analysis

Results are expressed as mean ± standard deviation (SD) or median (interquartile range). After checking for normality with Shapiro-Wilk, data were analyzed by a Kruskal-Wallis (continuous) or Chi-Square (categorical) test in case of the patient characteristics and parametric (t-test) or non-parametric (Mann-Whitney) test, as appropriate for the *in vitro* experiments. Statistical analyses were performed using SPSS (Statistical Package for Social Sciences), version 20.0, for Windows. Results with p-values below 0.05 were considered significant. Figures were produced using SPSS and GraphPad Prism 4 for Windows.

## Supplementary information

Supplementary Figure 1

## Data Availability

All data related to experiments presented are available upon request.
