# Supplementary material for: Contribution of the uremic milieu to an increased pro-inflammatory monocytic phenotype in chronic kidney disease
Source: Sci Rep. 2019 Jul 15;9:10236. doi: 10.1038/s41598-019-46724-5 (PMC6629661; doi:10.1038/s41598-019-46724-5)
Supplement: Supplementary file 1 — Supplementary Figure 1 [file 41598_2019_46724_MOESM1_ESM.pdf]

Contribution of the uremic milieu to an increased pro-inflammatory monocytic phenotype in chronic kidney disease.

Natalia Borges Bonan, Eva Schepers, Roberto Pecoits-Filho, Annemieke Dhondt, Anneleen Pletinck, Filip De Somer, Raymond Vanholder, Wim Van Biesen, Andréa Moreno-Amaral, Griet Glorieux.

Supplementary Figure 1

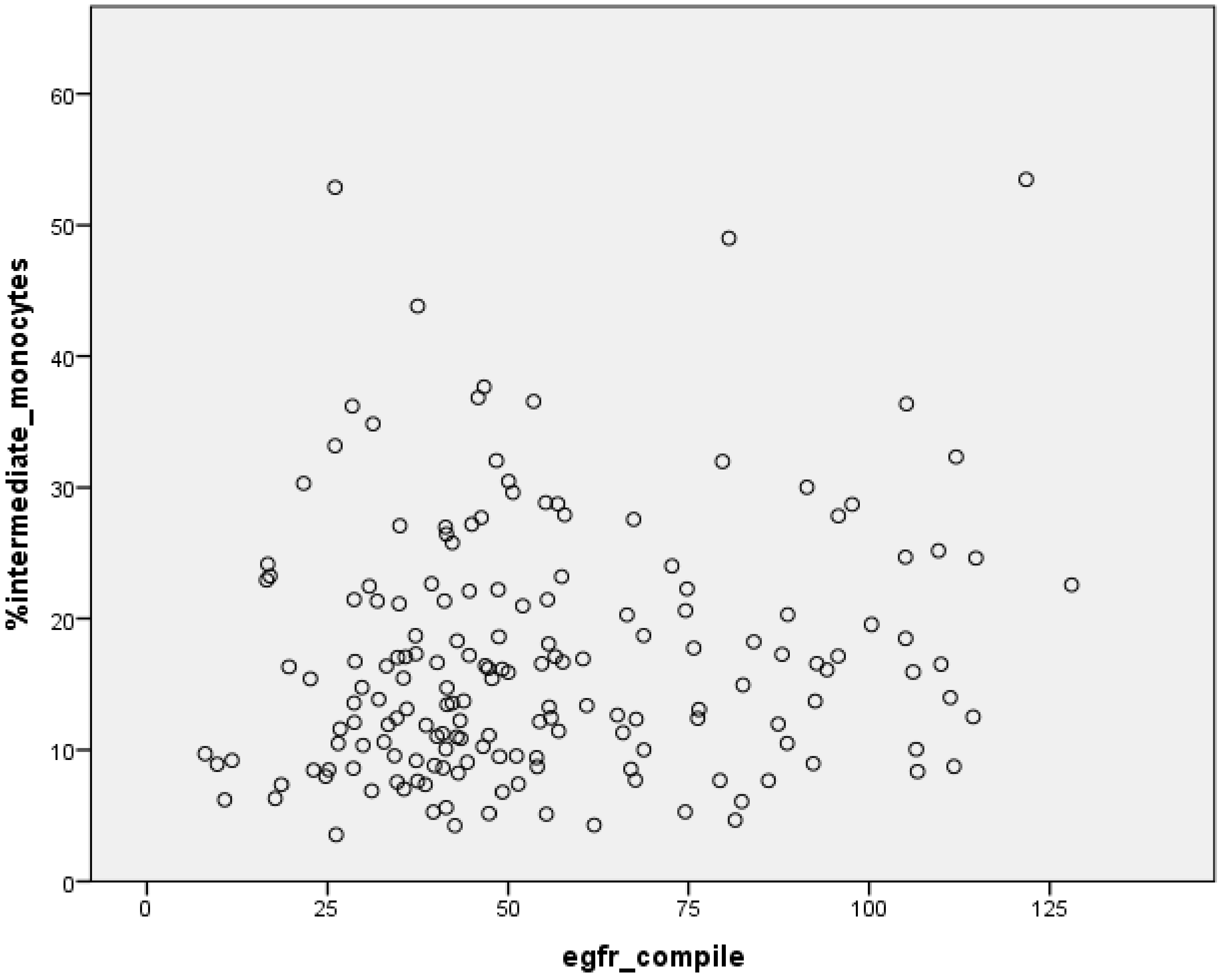

Supplementary Figure 1: Spearman's correlation between eGFR and the percentage intermediate ($CD14^{++}CD16^{+}$) monocytes in CKD patients not on dialysis. Correlation coefficient 0.157; P=0.039.
